# An Interesting Operation

**Published:** 1890-09

**Authors:** George W. Warren


### AN INTERESTING OPERATION.

BY GEORGE W. WARREN.

Mrs. M., twenty-eight years of age, presented herself for treatment at the clinic of the Pennsylvania College of Dental Surgery. She had worn an upper artificial denture for a number of years, which had been comfortable up to a few weeks prior to her visit to the college, when, as she expressed it, "a piece of her jaw-bone commenced to work through." Upon examination, it was found that at least an inch and a half of the superior maxillary bone was necrosed and entirely denuded. The disease was, from the family history, evidently the result of a scrofulous affection, and had attacked the bone at the median line and was following the ridge over the left side of the mouth. It was decided to remove the necrosed portion of the jaw at once, though nature had made no effort to separate it from the healthy bone, except at the median line. The operation was performed before the class by Dr. Warren. An incision was made in the soft tissue, which was laid back until healthy bone was reached; a deep groove was then cut on all sides of the necrosed portion,—the "New Cord Engine" being employed for this purpose. This was followed by a skilful manipulation of the chisel and mallet, after which the bone was removed with the forceps. When it was found that the left cuspid tooth was lying horizontal, on a line with the ridge, in the body of the healthy bone, this was also removed; the edges of the bone were then smoothed with the chisel, and the cavity, after being thoroughly cleansed, was packed with iodoform gauze, and the patient directed to use listerine daily as an antiseptic mouth-wash. This was continued for about a week, when the gauze was removed, the cavity cleansed with bichloride of mercury, and the edges of the soft tissue, which had been previously refreshened, were brought together and united by means of a stitch. The embedded tooth had without doubt induced the disease in this locality.

